# Differential Effects of Amount, Intensity, and Mode of Exercise Training on Insulin Sensitivity and Glucose Homeostasis: A Narrative Review

**DOI:** 10.1186/s40798-022-00480-5

**Published:** 2022-07-14

**Authors:** Katherine A. Collins, Leanna M. Ross, Cris A. Slentz, Kim M. Huffman, William E. Kraus

**Affiliations:** Duke Molecular Physiology Institute, Duke University School of Medicine, Durham, NC USA

**Keywords:** Type 2 diabetes mellitus, Prediabetes, Cardiometabolic health, Aerobic exercise, Resistance exercise, Intervention, Lifestyle medicine, Calorie restriction, Weight loss, Glycemic control

## Abstract

As type 2 diabetes remains a leading cause of morbidity and mortality, identifying the most appropriate preventive treatment early in the development of disease is an important public health matter. In general, lifestyle interventions incorporating exercise and weight loss via caloric restriction improve cardiometabolic risk by impacting several key markers of insulin sensitivity and glucose homeostasis. However, variations in the effects of specific types of exercise interventions on these markers have led to conflicting results surrounding the optimal amount, intensity, and mode of exercise for optimal effects. Moreover, the addition of weight loss via caloric restriction to exercise interventions appears to differentially impact changes in body composition, metabolism, and insulin sensitivity compared to exercise alone. Determining the optimal amount, intensity, and mode of exercise having the most beneficial impact on glycemic status is both: (1) clinically important to provide guidelines for appropriate exercise prescription; and (2) physiologically important to understand the pathways by which exercise—with and without weight loss—impacts glycemic status to enhance precision lifestyle medicine. Thus, the purposes of this narrative review are to: (1) summarize findings from the three Studies of a Targeted Risk Reduction Intervention through Defined Exercise (STRRIDE) randomized trials regarding the differential effects of exercise amount, intensity, and mode on insulin action and glucose homeostasis markers; and (2) compare the STRRIDE findings to other published dose–response exercise trials in order to piece together the various physiologic pathways by which specific exercise interventions—with or without weight loss—impact glycemic status.

**Key Points**

1. Lifestyle interventions including exercise and weight loss via caloric restriction improve cardiometabolic disease risk by impacting several key measures of insulin sensitivity and glucose homeostasis. However, variations in the effects of specific types of exercise interventions—with and without weight loss—on these measures have led to conflicting results surrounding the optimal amount, intensity, and mode of exercise for optimal effects.
2. Findings from the dose–response exercise trial literature indicate: (1) exercise interventions incorporating aerobic and resistance training or aerobic plus weight loss via caloric restriction produce the greatest beneficial effects on markers of insulin sensitivity and glucose homeostasis; (2) when matched for amount of energy expenditure relative to body weight, moderate intensity aerobic exercise elicits greater benefits for IVGTT-derived skeletal muscle insulin sensitivity and early phase pancreatic *β*-cell function, as well as OGTT-derived glucose tolerance; and (3) compared to a more moderate-intensity prescription, higher-intensity exercise appears to have a more potent effect on peripheral insulin sensitivity assessed during hyperinsulinemic–euglycemic clamps.
3. Further research encompassing rigorously designed dose–response exercise trials is needed to help determine the optimal lifestyle intervention prescriptions for prevention and treatment of type 2 diabetes.

## Introduction

As of 2017, type 2 diabetes impacts an estimated 451 million adults worldwide, with approximately 79% of those impacted living in low- and middle-income countries [1]. In the USA, type 2 diabetes is prevalent among an estimated 34.2 million individuals [2] with disproportionate effects on sedentary individuals with overweight or obesity [3, 4]. Type 2 diabetes arises from multiple disturbances in glucose homeostasis, including: (1) impaired insulin secretion; (2) insulin resistance, where cells throughout the body have an impaired ability to respond to insulin (i.e., decreased sensitivity); and (3) abnormalities in splanchnic glucose uptake [5, 6]. Moreover, after these disturbances manifest as impaired glucose tolerance or impaired fasting glucose—a prediabetic state—glucose homeostasis becomes more challenging to regain [7, 8]. Therefore, identifying the most appropriate preventive treatment—such as the lifestyle interventions exercise and diet—is an important public health issue.

In general, weight loss and exercise improve cardiometabolic risk profiles by impacting several markers of insulin sensitivity and glucose homeostasis. However, variations in the effects of specific types of exercise interventions on these markers have led to conflicting results surrounding the optimal amount and intensity of exercise for optimal effects across the cardiometabolic disease spectrum [9–17]. Furthermore, exercise interventions including weight loss via caloric restriction differentially effect changes in body composition, metabolism, and insulin sensitivity compared to interventions employing exercise alone [18–20]. Thus, determining the optimal amount, intensity, and mode of exercise training having the greatest impact on glycemic status is both: (1) clinically important to provide guidelines for appropriate exercise prescription; and (2) physiologically important to understand the pathways by which exercise—with and without weight loss—impacts glycemic status to enhance our ability to personalize these exercise prescriptions.

To delve deeper into the patterns of exercise–dose response on glycemic control, we acknowledge the complexity of disentangling the different mechanisms of action—many of which are largely unknown—among various fasting and dynamic markers of glucose control. In terms of fasting measures, both the homeostatic model assessment of insulin resistance (HOMA-IR) and fasting glucose are reflective of hepatic insulin sensitivity [6]. When the liver becomes insulin resistant, impairments arise in both glycogen synthesis and suppression of glucose production, as well as increases in lipogenesis and inflammatory protein synthesis [21]. On the other hand, the Lipoprotein Insulin Resistance Index (LP-IR)—a composite marker of six lipoprotein subclass and size parameters—appears to reflect adipose tissue insulin sensitivity, where the presence of high insulin levels impairs the suppression of lipolysis [22]. Suppressed lipolysis leads to elevated levels of free fatty acids, which can impair muscle signaling, promote hepatic gluconeogenesis, and impair glucose-stimulated insulin response [23–28].

The dynamic intravenous glucose tolerance test (IVGTT)-derived measures of acute insulin response to glucose (AIRg) and insulin sensitivity index (Si) represent pancreatic and skeletal muscle insulin sensitivity, respectively. In the early stages of insulin resistance, pancreatic *β*-cells compensate for hyperglycemia by oversecreting insulin. As the progression to diabetes continues, *β*-cell compensation is unable to keep up with the metabolic demands and fails to secrete sufficient insulin. When skeletal muscles become insulin resistant, impairments occur in insulin-stimulated glucose transport and glucose phosphorylation as well as reductions in glucose oxidation and glycogen synthesis. Skeletal muscle insulin resistance is also related to increased intramyocellular fat content and fatty acid metabolites, which may be attributed to defects in skeletal muscle mitochondrial oxidative phosphorylation [29]. Disposition index (DI)—the product of AIRg and Si—is an indirect marker of whether the level of insulin secretion is appropriate for the level of insulin resistance; therefore, DI provides an integrative assessment of early phase *β*-cell responses. In addition, the hyperinsulinemic–euglycemic clamp-derived measure of whole-body insulin-stimulated glucose utilization rate also reflects hepatic and skeletal muscle function as a marker of peripheral insulin sensitivity [30, 31]. Finally, oral glucose tolerance test (OGTT)-derived measures of insulin sensitivity—including Matsuda index, glucose area under the curve (AUC), and insulin AUC—are considered to reflect even more complex, integrated physiology mechanisms, encompassing not only hepatic and muscle insulin sensitivity, but also the effects of postprandially released gut hormones (e.g., the incretins glucose-dependent insulinotropic polypeptide and glucagon-like peptide-1), neurotransmitters, and gastric emptying [32, 33].

The three Studies of a Targeted Risk Reduction Intervention through Defined Exercise (STRRIDE) randomized trials were designed to understand the dose–response and mode specificity effects of exercise on reductions in cardiometabolic risk in participants with dyslipidemia (STRRIDE I and STRRIDE AT/RT) and prediabetes (STRRIDE-PD). Within each STRRIDE trial, intervention groups were matched on one of the key exercise parameters—amount, intensity, or mode—while varying the other parameters. In addition, STRRIDE-PD included an intervention group similar to the lifestyle arm of the Diabetes Prevention Program, which combined aerobic exercise with diet and weight loss. Collectively, the STRRIDE trials provide a unique opportunity to assess the effects of ten different exercise interventions on markers of cardiometabolic health. In this narrative review, we will summarize the findings from the STRRIDE trials surrounding the differential effects of amount, intensity, and mode of exercise on measures of insulin sensitivity and glucose homeostasis. Further, to piece together the various physiological pathways by which specific exercise interventions—with or without weight loss—impact glycemic status, we will compare STRRIDE findings to other published dose–response exercise trials with main outcomes related to glycemic control.

## Background and Methods

### The STRRIDE Randomized Clinical Trials

A detailed description of the three STRRIDE clinical trials has been presented elsewhere [34–36]. A total of 475 participants who completed the three trials and had pre- and post-intervention data available for markers of insulin sensitivity and glucose homeostasis were included in this narrative review. Participants with dyslipidemia [STRRIDE I (*n* = 237) and STRRIDE AT/RT (*n* = 125)] or prediabetes [STRRIDE-PD (*n* = 150)] were randomized to either control group or one of ten interventions (Table 1):

**Table 1:** Exercise intervention groups in the STRRIDE trials

Study and intervention group	*n*	Exercise prescription	
*STRRIDE I*			
High Amount/Vigorous Intensity	64	23 KKW	65–80% peak V̇O_2_
Low Amount/Vigorous Intensity	58	14 KKW	65–80% peak V̇O_2_
Low Amount/Moderate Intensity	57	14 KKW	40–55% peak V̇O_2_
*STRRIDE AT/RT*			
Aerobic Training (Low Amount/Vigorous Intensity)	47	14 KKW	65–80% peak V̇O_2_
Resistance Training	51	3 days/week, 3 sets/day, 8–12 reps of 8 exercises	
Aerobic + Resistance Training	44	14 KKW at 65–80% peak V̇O_2_ + 3 days/week, 3 sets/day, 8–12 reps of 8 exercises	
*STRRIDE-PD*			
High Amount/Vigorous Intensity	38	16 KKW	65–80% V̇O_2_ reserve
High Amount/Moderate Intensity	40	16 KKW	40–55% V̇O_2_ reserve
Low Amount/Moderate Intensity	35	10 KKW	40–55% V̇O_2_ reserve
Clinical Lifestyle Intervention	37	10 KKW at 40–55% V̇O_2_ reserve + caloric restriction to reduce body weight by 7%	

KKW, kcal exercise energy expenditure/kilogram of body weight/week; 23 KKW, calorically equivalent of walking/jogging approximately 20 miles/week for a 90 kg person; 14 KKW, calorically equivalent of walking/jogging approximately 12 miles/week for a 90 kg person; 16 KKW, calorically equivalent of walking/jogging approximately 13.8 miles/week for a 90 kg person; 10 KKW, calorically equivalent of walking/jogging approximately 8.6 miles/week for a 90 kg person

#### STRRIDE I (8-Month Intervention Duration; Tables 1 and 2)

**Table 2:** Baseline and change scores for fasting and IVGTT parameters in STRRIDE I by group

	Control (*n* = 58)			Low amount/moderate intensity (*n* = 57)			Low amount/vigorous intensity (*n* = 58)			High amount/vigorous intensity (*n* = 64)		
	Baseline	Change	*p*	Baseline	Change	*p*	Baseline	Change	*p*	Baseline	Change	*p*
*Fasting parameters*												
Glucose (mmol/L)	5.0 (0.6)	0.2 (0.5)	**	5.1 (0.5)	− 0.01 (0.5)	NS	5.1 (0.5)	0.1 (0.5)	NS	5.2 (0.6)	0.03 (0.5)	NS
Insulin (pmol/L)	51.0 (28.2)	5.4 (19.8)	*	61.8 (48.6)	− 15.0 (33.6)	**	52.8 (24.6)	− 9.0 (24.6)	**	57.6 (19.8)	− 7.8 (19.8)	**
HOMA-IR	1.1 (0.6)	0.1 (0.4)	*	1.3 (1.0)	− 0.3 (0.7)	**	1.2 (0.7)	− 0.2 (0.5)	**	1.2 (0.7)	− 0.2 (0.4)	**
LP-IR^a^	53.1 (25.2)	− 0.3 (17.4)	NS	60.0 (25.0)	− 9.3 (15.5)	***	52.1 (25.4)	− 5.2 (15.9)	*	54.7 (24.3)	− 6.1 (13.8)	**
DRI^a^	39.9 (21.3)	− 0.2 (12.0)	NS	43.7 (18.3)	− 4.7 (14.4)	*	43.0 (20.0)	− 2.6 (8.9)	NS	44.2 (20.4)	− 1.5 (12.2)	NS
*IVGTT parameters*												
DI	1562 (1444)	− 65 (893)	NS	1270 (1147)	742 (1680)	**	1533 (1362)	255 (1023)	NS	1305 (1121)	255 (685)	**
AIRg (mU/L/min)	490.6 (431.2)	22.1 (232.6)	NS	457.3 (334.1)	− 9.8 (207.3)	NS	445.5 (352.7)	− 42.8 (216.4)	NS	505.6 (426.6)	− 76.6 (217.6)	**
Si (mU/L/min)	3.5 (2.1)	− 0.4 (1.7)	NS	3.1 (2.2)	1.7 (2.5)	***	3.7 (2.1)	0.8 (1.8)	**	3.2 (2.5)	1.2 (2.1)	***

Data presented as means (SD)

AIRg, acute insulin response to intravenous glucose; DI, disposition index; DRI, Diabetes Risk Index; HOMA, homeostatic model assessment [(fasting glucose × fasting insulin)/22.5]; IVGTT, intravenous glucose tolerance test; LP-IR, Lipoprotein Insulin Resistance Index; NS, not significant; Si, insulin sensitivity index

*p < 0.05; **p < 0.01; ***p < 0.001

^a^LP-IR and DRI sample sizes are: Con (*n* = 53); Low/Mod (*n* = 44); Low/Vig (*n* = 48); High/Vig (*n* = 49)

1. High-amount/vigorous-intensity aerobic training: 23 kcal of exercise expenditure/kg of body weight/week (KKW) at 65–80% peak V̇O_2_
2. Low-amount/vigorous-intensity aerobic training: 14 KKW at 65–80% peak V̇O_2_
3. Low-amount/moderate-intensity aerobic training: 14 KKW at 40–55% peak V̇O_2_

#### STRRIDE AT/RT (8-Month Intervention Duration; Tables 1 and 3)

**Table 3:** Baseline and change scores for fasting and IVGTT parameters in STRRIDE AT/RT by group

	Aerobic training			Resistance training			Aerobic + resistance training		
	Baseline	Change	*p*	Baseline	Change	*p*	Baseline	Change	*p*
*n*	42			43			40		
*Fasting Parameters*									
Glucose (mmol/L)	5.3 (0.7)	− 0.1 (0.6)	NS	5.5 (0.6)	− 0.03 (0.5)	NS	5.1 (0.5)	0.01 (0.5)	NS
Insulin (pmol/L)	56.4 (33.6)	− 10.2 (18.0)	***	51.6 (22.8)	− 2.4 (28.2)	NS	52.8 (27.0)	− 9.6 (26.4)	*
HOMA-IR	2.3 (1.5)	− 0.5 (0.9)	**	2.1 (1.1)	− 0.1 (1.3)	NS	2.0 (1.1)	− 0.4 (1.1)	*
LP-IR^a^	54.0 (25.5)	− 4.8 (17.2)	NS	48.7 (24.4)	− 1.7 (14.0)	NS	54.3 (20.4)	− 10.1 (16.8)	***
DRI^a^	45.9 (19.2)	− 2.9 (11.9)	NS	40.8 (18.2)	− 0.8 (12.2)	NS	46.0 (16.3)	− 6.2 (11.0)	***

*n*	27			38			23		
*IVGTT parameters*									
DI	1813 (1341)	− 230 (1047)	NS	1794 (1204)	− 114 (1107)	NS	1465 (1192)	1069 (1696)	**
AIRg (mU/L/min)	471 (352)	− 103 (186)	**	495 (322)	− 35 (224)	NS	510 (449)	− 80 (248)	NS
Si (mU/L/min)	4.46 (3.1)	0.20 (2.8)	NS	4.08 (1.9)	− 0.21 (2.0)	NS	4.08 (1.9)	3.06 (3.4)	***

Data presented as means (SD)

AIRg, acute insulin response to intravenous glucose; DI, disposition index; DRI, Diabetes Risk Index; HOMA, homeostatic model assessment [(fasting glucose x fasting insulin)/22.5]; IVGTT, intravenous glucose tolerance test; LP-IR, Lipoprotein Insulin Resistance Index; NS, not significant; Si, insulin sensitivity index

*p < 0.05; **p < 0.01; ***p < 0.001

^a^LP-IR and DRI sample sizes are: aerobic training (*n* = 46); resistance training (*n* = 50); aerobic + resistance training (*n* = 43)

4. Resistance training only: 3 sets/day, 8–12 repetitions/set, of 8 exercises, 3 days/week
5. Aerobic training only (low amount/vigorous intensity): 14 KKW at 75% peak V̇O_2_
6. Aerobic plus resistance training: full combination of the low-amount/vigorous-intensity aerobic and resistance training prescriptions

#### STRRIDE-PD (6-Month Intervention Duration; Tables 1 and 4)

**Table 4:** Baseline and change scores for fasting and OGTT parameters in STRRIDE-PD by group

	Low amount/moderate intensity (*n* = 35)			High amount/moderate intensity (*n* = 40)			High amount/vigorous intensity (*n* = 38)			Clinical lifestyle (*n* = 37)		
	Baseline	Change	*p*	Baseline	Change	*p*	Baseline	Change	*p*	Baseline	Change	*p*
*Fasting parameters*												
Glucose (mmol/L)	5.87 (0.6)	0.05 (0.3)	NS	5.91 (0.5)	− 0.07 (0.4)	NS	5.79 (0.5)	0.06 (0.4)	NS	5.86 (0.6)	− 0.32 (0.4)	***
Insulin (pmol/L)	48.4 (24)	− 5.4 (16)	NS	45.3 (31)	− 6.4 (24)	NS	49.8 (44)	− 9.3 (24)	*	50.0 (28)	− 21.5 (22)	***
HOMA-IR	1.9 (1.0)	− 0.2 (0.7)	NS	1.8 (1.3)	− 0.3 (1.0)	NS	2.0 (1.9)	− 0.4 (1.0)	*	2.0 (1.2)	− 0.9 (0.9)	***
LP-IR^a^	54.5 (22.4)	− 0.6 (13.9)	NS	55.2 (22.6)	− 4.4 (16.2)	NS	55.2 (22.4)	− 4.4 (8.2)	**	57.1 (18.3)	− 12.4 (14.1)	***
DRI^a^	37.9 (16.8)	− 0.6 (10.0)	NS	38.5 (18.3)	− 1.4 (11.5)	NS	39.1 (17.1)	− 2.8 (8.2)	*	39.2 (16.8)	− 8.3 (10.4)	***
*OGTT parameters*												
Glucose AUC (mmol/L × 120 min)	1039 (219)	− 49 (143)	NS	1042 (192)	− 73 (123)	***	1031 (211)	− 22 (103)	NS	1050 (232)	− 96 (132)	***
Insulin AUC (pmol/L × 120 min)	955 (645)	− 166 (330)	**	990 (666)	− 264 (452)	***	1135 (999)	− 246 (452)	**	982 (527)	− 348 (350)	***
Matsuda Index	5.14 (3.2)	1.08 (1.7)	***	5.55 (3.5)	1.35 (2.7)	**	5.25 (3.0)	1.38 (2.4)	***	4.99 (3.3)	3.98 (3.9)	***

Data presented as means (SD)

AUC, area under the curve; DRI, Diabetes Risk Index; HOMA, homeostatic model assessment [(fasting glucose x fasting insulin)/22.5]; NS, not significant; OGTT, oral glucose tolerance test; LP-IR, Lipoprotein Insulin Resistance Index

*p < 0.05; **p < 0.01; ***p < 0.001

^a^LP-IR and DRI sample sizes are: Low/Mod (*n* = 42); High/Mod (*n* = 45); High/Vig (*n* = 40); CL (*n* = 43)

7. Low-amount/moderate-intensity aerobic training: 10 KKW at 50% V̇O_2_ reserve
8. High-amount/moderate-intensity aerobic training: 16 KKW at 50% V̇O_2_ reserve
9. High-amount/vigorous-intensity aerobic training: 16 KKW at 75% V̇O_2_ reserve
10. Clinical lifestyle intervention: low-amount/moderate-intensity aerobic training prescription plus a calorie restricted diet designed to reduce body weight by 7%

Insulin sensitivity for STRRIDE I and AT/RT was determined using a three-hour IVGTT [37]. Through an intravenous catheter placed in the antecubital space, glucose (50% at 0.3 g/kg body mass) was injected at time zero and insulin (0.025 U/kg body mass) was injected at *minute 20.* Twenty-nine blood samples (at *minutes 0, 2, 3, 4, 5, 6, 8, 10, 12, 14, 16, 19, 22, 23, 24, 25, 27, 30, 40, 50, 60, 70, 80, 90, 100, 120, 140, 160, 180*) were obtained, centrifuged, and stored at – 80 °C. Insulin was measured by immunoassay (Access Immunoassay System, Beckman Coulter, Fullerton, CA), and glucose with an oxidation reaction (YSI 2300, Yellow Springs, OH). Si, AIRg (calculated as area under the insulin curve during the first 10 min; a measure of insulin secretion), and DI (DI = AIRg X Si; a measure of *β*-cell function) were calculated using Bergman’s minimal model [37]. The IVGTT was performed after an overnight fast both at baseline and at the end of exercise training (16–24 h after the last exercise session).

Insulin sensitivity for STRRIDE-PD was determined using a two-hour OGTT. Participants drank a 75 g glucose drink with blood samples taken at 0, 30, 60, 90, and 120 min. Glucose was measured with a Beckman Coulter DxC600 clinical analyzer (Brea, CA, USA). Insulin was measured by electrochemiluminescent plate assay (Meso Scale Discovery, Gaithersburg, MD, USA). Glucose and insulin AUCs were calculated by the trapezoid method. Matsuda index was calculated as described in Matsuda and Defronzo [38]. The OGTT was performed after a 10-h fast both at baseline and at the end of exercise training (16–24 h after the last exercise session).

For all three STRRIDE studies, fasted plasma samples were analyzed on 400 MHz nuclear magnetic resonance profilers at LipoScience, now LabCorp (Morrisville, NC, USA), as previously described [39]. The lipoprotein parameters as well as the branched chain amino acids were calculated by retrospectively analyzing digitally stored spectra using the newly developed LP4 algorithm [40–43]. As previously described [44], LP-IR is a composite index calculated from the results of the following six lipoprotein parameters: large very low-density lipoprotein, small low-density lipoprotein, and high-density lipoprotein subclass concentrations and very low-density lipoprotein, low-density lipoprotein, and high-density lipoprotein sizes. LP-IR scores range from 0 (most insulin sensitive) to 100 (most insulin resistant). The Diabetes Risk Index is a multi-marker index composed of LP-IR, valine, and leucine. As described previously [45], the Diabetes Risk Index was developed using logistic regression and prospective type 2 diabetes data from the Multi-Ethnic Study of Atherosclerosis (MESA) [46]. Diabetes Risk Index scores range from 1 to 100, the latter representing those at greatest risk for type 2 diabetes. Further among fasting samples, HOMA-IR was calculated using fasting glucose multiplied by fasting insulin and divided by 22.5 as an indicator of insulin sensitivity during fasting conditions.

### Literature Search Procedures

The literature was reviewed to identify studies investigating the effects of exercise amount, intensity, and mode on insulin sensitivity and glucose homeostasis. The online database PubMed (MEDLINE) was searched between May 2020 and September 2021. We utilized a variety of MeSH terms and text words to narrow our search by population/problem (e.g., prediabetes, cardiovascular diseases), intervention (e.g., exercise training, lifestyle intervention), outcomes/measures (e.g., blood glucose, insulin sensitivity, glucose tolerance), and sources (e.g., controlled clinical trial). Study inclusion was limited to randomized controlled trials only.

## Aerobic Exercise Amount and Intensity Effects

### STRRIDE Findings

Among traditional fasting makers of glycemic status, all exercise training groups in STRRIDE I significantly improved fasting insulin (all exercise groups: − 7.8 ± 19.8 to − 15.0 ± 33.6 pmol/L, *p* < 0.01) and HOMA-IR (all exercise groups: − 0.2 ± 0.4 to − 0.3 ± 0.7, *p* < 0.01) (Table 2). However, no STRRIDE I exercise training groups significantly improved fasting glucose. Within the spectroscopically derived markers of insulin resistance and type 2 diabetes risk assessed in STRRIDE I, all three exercise groups significantly improved LP-IR (all exercise groups: − 5.2 ± 15.9 to − 9.3 ± 15.5, *p* < 0.05). Both the low-amount/moderate-intensity (− 4.7 ± 14.4) and low-amount/vigorous-intensity (− 2.6 ± 8.9) groups improved Diabetes Risk Index; however, these changes were only statistically significant for the low-amount/moderate-intensity group (*p* < 0.05). In STRRIDE-PD, both high amount exercise groups had similar magnitudes of change in LP-IR; however, only the high-amount/vigorous-intensity group (− 4.4 ± 8.2, *p* < 0.01) achieved statistical significance. The high-amount/vigorous-intensity group (− 2.8 ± 8.2, *p* < 0.05) experienced a significant decrease in Diabetes Risk Index, with no significant effect seen in the moderate-intensity groups (Table 4).

**Fig. 1:**
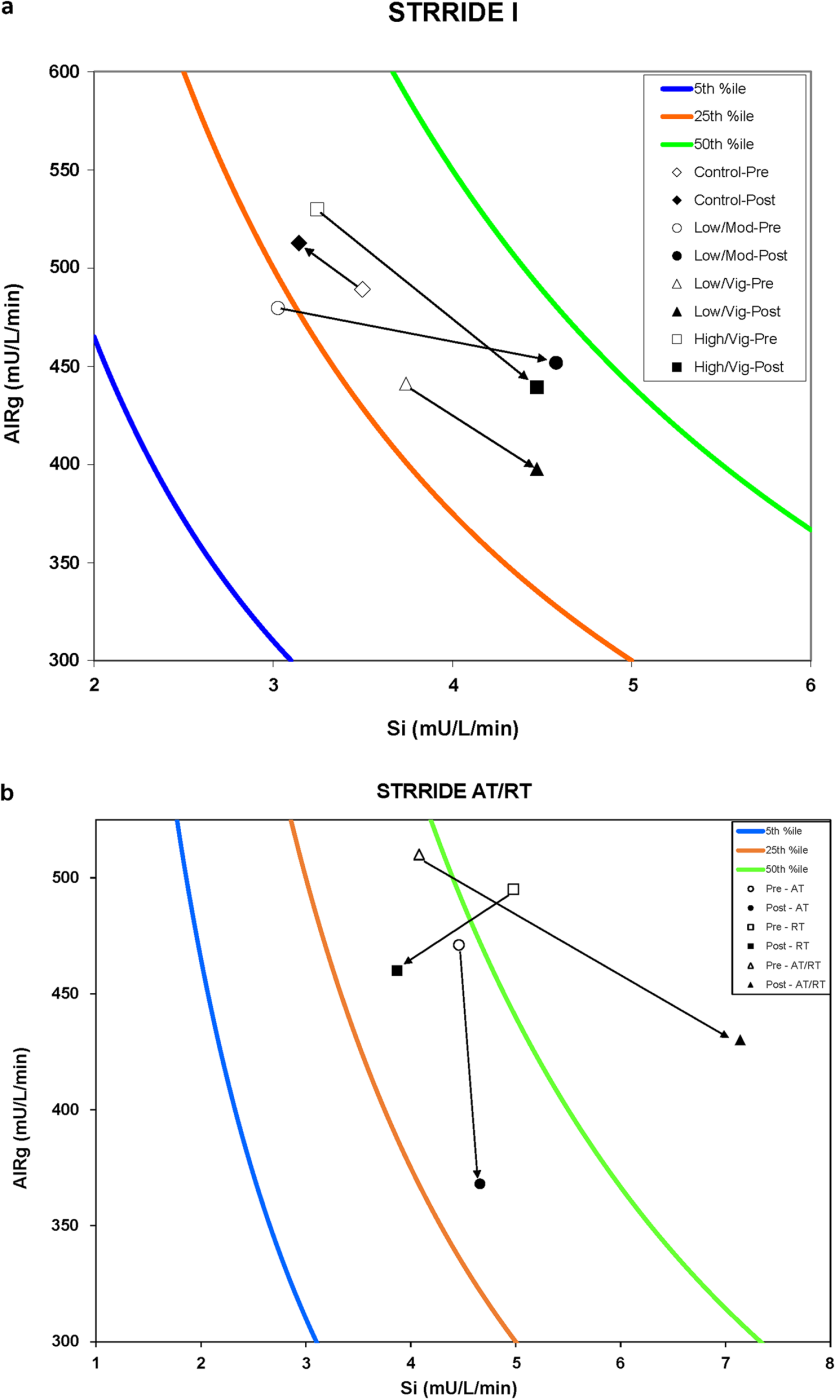
Disposition Index (DI = Si × AIRg) percentiles for STRRIDE I (panel **a**) and STRRIDE AT/RT (panel **b**). Type 2 diabetes risk is significantly modified only by changes across the isobar (DI) lines, with a greater percentile being representative of greater insulin sensitivity and lesser diabetes risk. Pre-intervention data points are represented by open symbols. Post-intervention data points are represented by closed symbols. Arrows represent the direction of change in DI following exercise training by intervention group. In panel **a**, diamonds = inactive control group; circles = low-amount/moderate-intensity group; triangles = low-amount/vigorous-intensity group; and squares = high-amount/vigorous-intensity group. In panel **b**, circles = aerobic training group; squares = resistance training group; and triangles = aerobic plus resistance training group. DI = disposition index; Si = insulin sensitivity index; AIRg = acute insulin response to glucose

Among IVGTT parameters, both moderate- and vigorous-intensity groups in STRRIDE I improved measures of insulin sensitivity and *β*-cell function (Table 2). Kahn and colleagues [47–50] have derived percentiles to assess the relationship between Si, AIRg, and DI, with significant diabetes risk being modified only by changes across the isobar (DI) lines. A lower percentile represents greater impairment in *β*-cell function and increased diabetes risk. Figure 1 Panel A shows the change in DI, Si, and AIRg for the STRRIDE I intervention groups. Compared to normal *β*-cell functioning individuals with a DI ranging from 2000 to 2800 [15, 51–53], STRRIDE I participants had a lower average baseline measure of pancreatic *β*-cell function, with a mean DI across intervention groups being approximately 1400. Following the intervention, DI significantly improved within the low-amount/moderate-intensity (742.1 ± 1680.0, *p* < 0.01) and high-amount/vigorous-intensity groups (254.5 ± 688.2, *p* < 0.01). All three exercise training groups experienced a significant improvement in Si (all exercise groups: 0.8 ± 1.8 to 1.7 ± 2.5 mU/L/min, *p* < 0.001). Only the high-amount/vigorous-intensity group significantly improved AIRg (-76.6 ± 217.6 pmol/l, *p* < 0.01). The magnitude of improvement in DI and Si among the low-amount/moderate-intensity group was greater than that for the same amount of exercise at a vigorous intensity (p < 0.05).

Among OGTT parameters in STRRIDE-PD, all exercise groups significantly improved insulin AUC (− 166.0 ± 330.0 to − 264.0 ± 452.0 pmol/L × 2 h, *p* < 0.01) and Matsuda index (1.1 ± 1.7 to 1.4 ± 2.4, *p* < 0.01). Of the exercise *without* diet groups, only the high-amount/moderate-intensity group significantly improved glucose AUC (− 73.0 ± 123.0 mmol/L × 120 min, *p* < 0.001), exceeding results from the group performing vigorous-intensity exercise of the same amount (Table 4). Consistent with STRRIDE I, these findings further support moderate—rather than vigorous—intensity exercise that has greater effects on measures of insulin sensitivity and glucose homeostasis.

### Previous Findings in the Literature

Few randomized controlled trials have rigorously compared amount and intensity effects on insulin action and glucose homeostasis measures; we subsequently discuss the major findings from four randomized controlled trials. Highlighted results from these studies are presented in Table 5.

**Table 5:** Summary of studies evaluating aerobic exercise amount and intensity effects on markers of insulin sensitivity and glucose homeostasis

Study	*n*	Population	Training type/intervention length	Groups	Measure	Results
DiPietro et al. [10]	25	BMI < 30 kg/m^2^ > 60 yrs Women	9-month aerobic intervention	*Vigorous Intensity*: 300 kcal/session at 80% peak V̇O_2_ *Moderate Intensity*: 300 kcal/session at 65% peak V̇O_2_ *Control/Low Intensity*: 45 min/session at 50% peak V̇O_2_	Hyperinsulinemic–euglycemic clamp	*Fasting Glucose* No change in exercise groups
						*Fasting Insulin* No change in exercise groups
						*Whole-Body Glucose Utilization Rate at 40 mU* Significant increase in Vig. Group
						*Basal Hepatic Glucose Production* Significant decrease in Mod. Group
Coker et al. [12]	21	BMI: ≥ 26 to < 37 kg/m^2^ 65 to 90 yrs	12-week aerobic intervention	*Vigorous Intensity*: 1000 kcal/week at 75% peak V̇O_2_ *Moderate Intensity*: 1000 kcal/week at 50% peak V̇O_2_ *No Exercise Control*		*Insulin-stimulated Glucose Disposal* Significant increase in Vig. Group
						*Non-oxidative Glucose Metabolism* Significant increase in Vig. Group
Ross et al. [54]	300	Abdominal obesity: Men WC > 102 cm or Women WC > 88 cm 35 to 65 yrs	24-week aerobic intervention	*No Exercise Control* *Low Amount/Moderate Intensity*: Women: 180 kcal/session and Men: 300 kcal/session at 50% peak V̇O_2_ *High Amount/Moderate Intensity*: Women: 360 kcal/session and Men: 600 kcal/session at 50% peak V̇O_2_ *High Amount/Vigorous Intensity*: Women: 360 kcal/session and Men: 600 kcal/session at 75% peak V̇O_2_	Oral glucose tolerance test	*2-h Glucose* Significant difference in change between High/Vig vs. Control Significant difference in change between High/Vig vs. Low/Mod
						*Glucose AUC* Significant difference in change between High/Vig vs. Low/Mod
						*Insulin AUC* Significant difference in change between High/Vig and High/Mod vs. Control
						*Matsuda Index* Significant difference in change between High/Vig and High/Mod vs. Control Significant difference in change between High/Vig vs. Low/Mod
Malin et al. [55]	31	Average BMI: 32.1 kg/m^2^ Average age 61.4 yrs Prediabetes Nonsmoking Physically inactive	13-day aerobic intervention	*Moderate Intensity/ Continuous Exercise:* 60 min each day at 70% peak HR; rest on day 7 *Vigorous Intensity/ Interval Exercise*: 60 min each day alternating 3-min intervals at 90% peak HR followed by 50% peak HR; rest on day 7	Oral glucose tolerance test	*Glucose AUC* Significant decrease in both groups
						*Insulin AUC* Significant decrease in both groups
						*Skeletal Muscle Disposition Index* Significant increase in both groups (early and total-phase)
						*Oral Minimal Model (Skeletal Muscle Insulin Sensitivity)* Significant increase in both groups

AUC, area under the curve; BMI, body mass index; HR, heart rate; WC, waist circumference

Ross and colleagues investigated the separate effects of exercise amount and intensity on changes in abdominal obesity and OGTT-derived two-hour glucose concentrations [54]. Rather than abnormal glucose homeostasis parameters, participants were initially recruited based on abdominal obesity criteria. Three hundred abdominally obese participants were randomized to either no exercise control or to one of three exercise groups performing five sessions per week for 24 weeks of (1) low-amount/low [moderate]-intensity exercise (180 and 300 kcal/session for women and men, respectively, at 50% of maximum oxygen consumption [peak V̇O_2_]); (2) high-amount/low [moderate]-intensity exercise (360 and 600 kcal/session for women and men, respectively, at 50% of peak V̇O_2_); or (3) high-amount/high [vigorous]-intensity exercise (360 and 600 kcal/session for women and men, respectively, at 75% of peak V̇O_2_). Of note, as indicated above, for this review, we define exercising at 50% peak V̇O_2_ to represent moderate intensity, rather than the study-specified “low” intensity. Primary outcome variables included waist circumference and two-hour glucose level, measured in response to a two-hour, 75 g OGTT at baseline and between 36 and 48 h after the last exercise session at 16 and 24 weeks. Compared to control, reductions in two-hour glucose level were greater in the high-amount/high [vigorous]-intensity group. While the authors concluded that high-amount/high-intensity exercise reduces two-hour glucose levels greater than high-amount/low [moderate]-intensity and low-amount/low [moderate]-intensity, significant group differences were not present. Compared to control, both the high-amount/low [moderate]-intensity and high-amount/high [vigorous]-intensity groups significantly improved Matsuda index and insulin AUC.

While holding absolute exercise volume—not relative to body weight—constant, DiPietro and colleagues studied the relative benefits of a moderate- versus high-intensity 9-month exercise training program on insulin sensitivity [10]. Twenty-five sedentary, older women were randomized to 4 days per week of either (1) high-intensity aerobic exercise (300 kcal/session at 80% of peak V̇O_2_); (2) moderate-intensity aerobic exercise (300 kcal/session at 65% peak V̇O_2_); or (3) low-intensity placebo control (45 min/session at 50% peak V̇O_2_). Whole-body insulin-stimulated glucose utilization was determined using a two-step euglycemic–hyperinsulinemic clamp, following methods described by DeFronzo et al. [30], at baseline and at 9 months, ~ 72 h following the last training session. Following a resting period, regular human insulin was infused as a primed continuous (120-min) low-dose infusion (10 mU m^−2^ min^−1^) followed by a continuous (120-min) higher dose infusion (hyperglycemic; 40 mU m^−2^ min^−1^) during which glucose (20% dextrose) infusion was adjusted at a rate to maintain plasma glucose at ~ 100 mg/dL during hyperinsulinemia. Following the intervention, no groups experienced a significant change in fasting plasma glucose, insulin, glycerol, or free fatty acid concentrations. Seventy-two hours after the last session of exercise training, the high-intensity group experienced a significant long-term training-related improvement in the rate of whole-body insulin-stimulated glucose utilization at the higher insulin dose. The high-intensity group also experienced significant improvements in insulin-stimulated suppression of adipose tissue lipolysis to the low dose of insulin as well as improvements in glucose uptake when normalized for the level of circulating insulin during the final 30 min of the clamp. These improvements appeared to follow a dose–response trend with regard to intensity; a greater magnitude of improvement was found among those in the high-intensity group compared to moderate- or low-intensity control. Further, these improvements were observed without improvements in body composition and peak V̇O_2_.

Adding to work from DiPietro and colleagues [10], Coker and colleagues examined the effects of moderate- versus high-intensity exercise training on insulin-stimulated glucose disposal, holding constant prescribed exercise volume [12]. Twenty-one overweight, older adults were randomized to perform aerobic exercise training 4 to 5 days per week for 12 weeks at either high-intensity exercise training (1000 kcal/week at 75% V̇O_2max_); moderate-intensity (1000 kcal/week at 50% V̇O_2max_); or to non-exercise control group. Insulin sensitivity was measured using a 120-min hyperinsulinemic–euglycemic clamp at baseline and post-intervention. Post-intervention clamps were completed 3 days after the last exercise session. Following the 16-week intervention, in the absence of weight loss, the moderate-intensity and control groups did not experience changes in insulin-stimulated glucose disposal, non-oxidative glucose metabolism, or glucose oxidation. On the other hand, the high-intensity group significantly improved insulin-stimulated glucose disposal, which was entirely reliant on an increase in non-oxidative glucose metabolism, seemingly reflecting the influence of muscle glycogen content on insulin sensitivity.

In a short-term exercise intervention, Malin and colleagues investigated the effect of amount-matched intensity exercise on *β*-cell function, adjusting for gut hormones and skeletal muscle insulin sensitivity, among individuals with prediabetes [55]. Thirty-one participants were randomized to 12 work-matched sessions over a 13-day period of either moderate-intensity continuous exercise [60 min/day at 70% peak heart rate (HR)] or high-intensity interval exercise (60 min/day alternating 3-min intervals at 90% peak HR followed by 50% peak HR). Early (0–30 min) and total-phase (0–120 min) glucose tolerance and pancreatic *β*-cell function were measured using a 75 g two-hour OGTT following an overnight fast at baseline and post-intervention. Following exercise training, both groups significantly improved early and total-phase glucose AUC, insulin AUC, and skeletal muscle DI, as well as skeletal muscle insulin sensitivity derived from the oral minimal model. Pearson’s correlation revealed a significant relationship between improvements in glucose AUC with increases in early and total-phase skeletal muscle DI. Only continuous moderate-intensity exercise training raised fasting glucose-dependent insulinotropic polypeptide, whereas both exercise intervention groups increased early phase glucagon-like polypeptide-1 during the OGTT. Overall, the authors concluded that in adults with prediabetes, *β*-cell function improved independent of exercise intensity, when adjusting for skeletal muscle insulin sensitivity. These findings suggest that exercise promotes a unique compensatory mechanism between skeletal muscle, gut, and pancreas to reduce ambient glucose concentration.

### Summary

Although discerning distinct dose–response effects of aerobic exercise across diverse markers of glycemic status remains difficult, some studies identified differential amount and intensity effects for certain markers. For example in STRRIDE I, both low-amount/moderate-intensity and high-amount/vigorous-intensity exercise elicited significant improvements in DI, with low-amount/moderate-intensity having the greatest effect—driven by substantial improvements in skeletal muscle insulin sensitivity (Si) and almost no compensatory decrease in first-phase insulin secretion (AIRg). Interestingly for the Si component, twice the amount of vigorous intensity exercise was necessary to elicit the same magnitude of response as the low-amount/moderate-intensity group. The beneficial effects of moderate-intensity exercise were also observed in STRRIDE-PD; when matched for amount relative to body weight, moderate intensity had a significantly superior effect on glucose tolerance (glucose AUC) compared to vigorous intensity exercise. Paradoxically, compared to a more moderate-intensity prescription, higher-intensity exercise appears to have a more potent effect on peripheral insulin sensitivity assessed during hyperinsulinemic–euglycemic clamps.

Notably, for three studies in this narrative review, aerobic exercise—regardless of amount and intensity—significantly improved a variety of insulin sensitivity and glucose homeostasis markers. In the two-week aerobic intervention study conducted by Malin et al., both exercise groups significantly improved OGTT-derived markers insulin AUC, glucose AUC, skeletal muscle insulin sensitivity, and skeletal muscle DI. After 6 months of aerobic training in STRRIDE-PD, all exercise groups significantly improved the OGTT-derived markers insulin AUC and Matsuda index. Following 8 months of aerobic training in STRRIDE I, all exercise groups significantly improved the fasting markers LP-IR, insulin, and HOMA-IR, and the IVGTT-derived marker Si.

Overall, aerobic exercise-mediated improvements occur through increased expression and activation of signaling proteins in the skeletal muscles—such as glucose transporter type 4 (GLUT4) translocation mediated by adenosine monophosphate-activated protein kinase (AMPK) and its downstream targets—involved in the regulation of glucose uptake and metabolism as well as increases in lipid turnover and oxidation [56, 57]. In addition, to help support the increased oxygen demands in the exercising muscles, aerobic training increases capillary density and promotes mitochondrial biogenesis [58]. Expanding the mitochondrial network improves skeletal muscle capacity for oxygen consumption and production of adenosine triphosphate (ATP) [58]. Aerobic exercise also decreases basal glucose production and increases suppression of liver glucose output [59].

Moderate- and vigorous-intensity aerobic exercises are well known to primarily rely on different sources of energy. Moderate-intensity exercise reflects a greater percentage of fat oxidation compared to vigorous intensity. Plausibly, the moderate-intensity-induced improvements in fat oxidation lead to a reduction in skeletal muscle, liver, and pancreas lipotoxicity, thus improving insulin sensitivity [15]. Conversely, vigorous-intensity exercise relies on non-oxidative metabolism to a greater degree than moderate-intensity exercise [60]. Therefore, the vigorous intensity-induced improvements in insulin-stimulated glucose disposal rate are likely explained by the greater depletion of muscle glycogen content necessitating a compensatory increase in muscle glycogen synthesis [12].

## Exercise Mode Effects

### STRRIDE Findings

Among traditional fasting measures of glycemic status in STRRIDE AT/RT, aerobic training—alone and in combination with resistance training—significantly improved both fasting insulin (aerobic only: − 10.2 ± 18.0 pmol/L, *p* < 0.001; aerobic plus resistance: − 9.6 ± 26.4 pmol/L, *p* < 0.05) and HOMA-IR (aerobic only: − 0.5 ± 0.9, *p* < 0.01; aerobic plus resistance: − 0.4 ± 1.1, *p* < 0.05) (Table 3). However, resistance training alone did not significantly improve any fasting measures. Further, none of the three exercise training groups significantly improved fasting glucose. For the novel markers of insulin resistance and type 2 diabetes risk, only the aerobic plus resistance training group resulted in a significant and robust decrease in LP-IR (− 10.1 ± 16.8, *p* < 0.001), which was significantly different from the resistance training only group. Similarly, only aerobic plus resistance training induced a significant beneficial change in Diabetes Risk Index (− 6.2 ± 11.0, *p* < 0.001) (Table 3).

Compared to STRRIDE I participants, baseline pancreatic *β*-cell function among STRRIDE AT/RT participants was greater, with an average DI of ~ 1700. Figure 1 Panel B shows the change in DI, Si, and AIRg for the STRRIDE AT/RT intervention groups. Among these IVGTT parameters, the aerobic plus resistance training group had robust, synergistic changes in DI (1069.0 ± 1696.0, *p* < 0.01) and Si (3.1 ± 3.4 mU/L/min, *p* < 0.001) compared to aerobic and resistance training alone (Table 3). Neither aerobic nor resistance training alone resulted in significant improvements in these measures. Although all three exercise training groups reduced AIRg, this change was only significant in the aerobic training only group (− 103.0 ± 186.0 mU/L/min, *p* < 0.01).

### Previous Findings in the Literature

Tremendous consistency exists in the literature regarding mode effects of exercise on insulin action and glucose homeostasis measures. The combination of aerobic and resistance training is superior to aerobic or resistance training alone for improving insulin sensitivity and glycemic control. Nevertheless, aerobic and resistance training alone lead to some moderate improvements in insulin sensitivity [13, 14, 61–73]. Highlighted results from the following studies evaluating exercise mode effects are presented in Table 6.

**Table 6:** Summary of studies evaluating exercise mode effects on markers of insulin sensitivity and glucose homeostasis

Study	*n*	Population	Training type/intervention length	Groups	Measure	Results
Sigal et al. (DARE Trial) [13]	251	39 to 70 yrs Type 2 diabetes Physically inactive	22-week aerobic and resistance intervention	*No Exercise Control* *Aerobic Training (Vigorous Intensity)*: 45 min/session at 75% maximum HR on 3 days/week *Resistance Training*: 2–3 sets of 7–9 repetitions of 7 exercises on 3 days/week *Aerobic* + *Resistance Training*: 45 min/session at 75% maximum HR + 2–3 sets of 7–9 repetitions of 7 exercises on 3 days/week	Turbidimetric immunioinhibition	*Hemoglobin A*_*1c*_ Significant difference in change between aerobic and resistance training groups vs. control group Significant difference in change between aerobic and resistance training groups vs. aerobic + resistance training group
Church et al. (HART-D Trial), [14]	262	30 to 75 yrs BMI < 48 kg/m^2^ Type 2 diabetes Sedentary	9-month aerobic and resistance intervention	*No Exercise Control* *Aerobic Training (Moderate-to-Vigorous Intensity)*: 12 KKW at 50–80% maximum V̇O_2_ *Resistance Training*: 2 sets of 4 upper body, 3 sets of 3 lower body, and 2 sets of 2 abdominal exercises of 10–12 repetitions on 3 days/week *Aerobic* + *Resistance Training*: 10 KKW at 50–80% maximum V̇O_2_ + 1 set of 10–12 repetitions of 9 exercises on 2 days/week	Automated glycosylated hemoglobin analyzer	*Hemoglobin A*_*1c*_ Significant decrease in Aerobic + Resistance training group Significant difference in change between aerobic and aerobic + resistance groups vs. control group when analysis population was limited to participants with a baseline hemoglobin A_1c_ ≥ 7.0%

AUC, area under the curve; HR, heart rate; KKW, kcal energy expenditure/kg of body weight/week

The Diabetes Aerobic and Resistance Exercise (DARE) trial aimed to determine the glycemic control effects of aerobic and resistance training alone versus a sedentary control group, and the incremental effects of performing both types of exercise (combined exercise training) versus aerobic or resistance training alone [13]. Two hundred fifty-one adults with type 2 diabetes were randomized to 22 weeks of no exercise control or 3 days per week of (1) aerobic training (45 min/session at 75% HR_max_), (2) resistance training (7 exercises/session, 2 to 3 sets of 7 to 9 repetitions per set), or (3) both aerobic and resistance training (45 min/session at 75% HR_max_ + 7 exercises/session, 2 to 3 sets of 7 to 9 repetitions per set). The primary outcome was absolute change from baseline to post-intervention in HbA_1c_ value, measured by turbidimetric immunoinhibition. The following results are adjusted estimated means from linear mixed-effects models. Compared to the control group, change in HbA1c was significantly greater in the aerobic training group (difference in change: − 0.51%). Similarly, as compared to control, change in HbA1c values was significantly greater in the resistance training group (difference in change: − 0.38%). For the combined exercise training group, change in HbA_1c_ values provided an additional 0.46% decrease compared to the aerobic training group and an additional 0.59% decrease compared with the resistance training group. Further, exercise-induced improvements in glycemic control were greater among participants with greater baseline HbA_1c_; among participants with lesser baseline HbA_1c_, only those in the combined exercise training group improved. Seemingly, individuals with type 2 diabetes and good glycemic control who wish to improve their HbA_1c_ through lifestyle change should perform both aerobic and resistance exercise. If glycemic control is poor, either aerobic or resistance training alone is suitable to improve HbA_1c_, but combination exercise training continues to be superior to either mode alone.

The goal of the Health Benefits of Aerobic and Resistance Training in individuals with type 2 diabetes (HART-D) trial was to compare aerobic training alone, resistance training alone, and a combination of both on HbA_1c_ in sedentary individuals, while maintaining similar weekly training durations—thus, the combination group performed approximately half the time of each mode compared to the aerobic and resistance training alone groups [14]. Two hundred sixty-two participants were randomized to a non-exercise control or (1) aerobic exercise training (12 KKW at 50 to 80% V̇O_2max_); (2) resistance exercise training only (3 days/week, 2 sets of 4 upper body, 3 sets of 3 lower body, and 2 sets of 2 abdominal exercises of 10 to 12 repetitions); or (3) combined resistance and aerobic training intervention (10 KKW at 50 to 80% V̇O_2max_ plus 2 sessions/week, performing 1 set of 9 exercises, at 10 to 12 repetitions). The primary outcome was HbA_1c_ measured during monthly visits. Following the intervention, only the combination group experienced a significant improvement in HbA_1c_ (− 0.23%). The combination group was significantly different compared to the control group (between-group difference in change: − 0.34%, *p* = 0.03). Neither resistance training nor aerobic training alone resulted in a significant change in HbA_1c_ compared to the control group. Thus, although both resistance and aerobic exercise training provide health benefits, only the combination of the two is associated with reductions in HbA_1c_ in individuals with type 2 diabetes. Moreover, the cumulative benefit among change in HbA_1c_ is superior in the combined exercise training group compared with either aerobic or resistance training alone. As in the STRRIDE AT/RT study, these findings appear to be more than additive effects when combining aerobic with resistance exercise on glucose control measures.

### Summary

Interestingly, for individuals with dyslipidemia in STRRIDE AT/RT, resistance training alone did not significantly impact fasting and IVGTT-derived markers of insulin sensitivity. For individuals with type 2 diabetes in DARE and HART-D, the distinct effects of resistance training alone on changes in HbA_1c_ were less clear. In DARE, compared to no exercise control, resistance training significantly reduced HbA_1c_, whereas in HART-D, there was no significant effect of resistance training on HbA_1c_. These discordant findings are likely attributable to differences in study design and population (e.g., resistance training prescriptions, duration of diabetes, and medications).

Resistance training is thought to increase GLUT4 expression in skeletal muscle and reduce the burden on pancreatic *β*-cells to secrete insulin [11, 56]. Moreover, resistance training is well known to increase protein synthesis and muscle fiber hypertrophy through the mechanistic target of rapamycin (mTOR) signaling pathway; however, mTOR’s role in improving insulin sensitivity remains unknown. In some studies, the failure of resistance training to improve insulin sensitivity in individuals with metabolic disturbances coincides with diminished phosphorylation of muscle AMPK and increased phosphorylation of mTOR [56]. These findings suggest that activation of the mTOR pathway may be involved in inhibition of exercise training-related increases in AMPK activation and its downstream targets.

When resistance training is paired with vigorous intensity aerobic exercise, marked improvements occur in Si, DI, LP-IR, fasting insulin, HOMA-IR, and HbA_1c_. The mechanisms by which combination training (*i.e.,* both aerobic and resistance exercise) elicits these notable improvements remain unknown. Of note, in both STRRIDE AT/RT and DARE, the participants in the combination groups exercised approximately double the time that the aerobic and resistance training alone groups performed. Therefore, we do not know if the marked beneficial combination training effects on insulin sensitivity in these two studies are due to the greater total volume of exercise performed or are due to a mechanistic synergy of the two exercise modes. However, findings from HART-D—where exercise volume was comparable between the combination, aerobic only, and resistance only groups—support the notion that combination training produces more than additive, even synergistic, effects for markers of glycemic status.

## Clinical Lifestyle Intervention Effects

### STRRIDE Findings

Within traditional fasting measures of glycemic status in STRRIDE-PD, the clinical lifestyle intervention—low-amount/moderate-intensity aerobic exercise with 7% weight loss goal via caloric restriction—improved both fasting insulin (− 21.5 ± 22.0 pmol/L, *p* < 0.001) and HOMA-IR (− 0.9 ± 0.9, *p* < 0.001) (Table 4). Further, the clinical lifestyle group was the only one to significantly improve fasting glucose (− 0.3 ± 0.4, *p* < 0.001). Among the spectroscopy-derived markers of insulin resistance and type 2 diabetes risk, the clinical lifestyle group had the most robust decrease in LP-IR (− 12.4 ± 14.1, *p* < 0.001); this change was significantly greater than all other exercise-only groups (*p* < 0.001 for difference among groups). Further, the clinical lifestyle intervention induced a significant improvement in Diabetes Risk Index (− 8.3 ± 10.4, *p* < 0.001), which was significantly greater than the change in both moderate-intensity exercise-only groups (*p* = 0.002 for difference among groups). Among OGTT measures, all exercise intervention groups significantly improved insulin AUC (ranging from − 348 ± 350 pmol/L × 2 h to − 166 ± 330 pmol/L × 2 h; *p* < 0.01 for all groups) and Matsuda Index (ranging from 1.08 ± 1.7 to 3.98 ± 3.9; *p* < 0.01). Only the high-amount/moderate-intensity (− 73 ± 123 mmol/L × 120 min, *p* < 0.001) and the clinical lifestyle (− 96.0 ± 132.0 mmol/L × 120 min, *p* < 0.001) groups improved glucose AUC.

### Previous Findings in the Literature

Lifestyle interventions including exercise in addition to caloric restriction resulting in weight loss—typically of at least 5% body weight—improve measures of insulin sensitivity and glucose homeostasis. Usually, these improvements out-perform interventions including only exercise; however, exercise alone in comparison with weight loss plus exercise still has a substantial effect on insulin action and glucose homeostasis [18–20, 74–81]. Highlighted results from the following studies evaluating lifestyle intervention effects are presented in Table 7.

**Table 7:** Summary of studies evaluating lifestyle intervention effects on markers of insulin sensitivity and glucose homeostasis

Study	*n*	Population	Training type/intervention length	Groups	Measure	Results
Cox et al. [18]	60	Average BMI 31.0 kg/m^2^ 20 to 50 yrs Men Nonsmoking Sedentary No substantial weight loss in previous 12 months	16-week caloric restriction and aerobic exercise intervention	*Maintain Diet* + *Control Exercise Group*: 30 min/session at light intensity on 3 days/week *Maintain Diet* + *Vigorous Intensity Group:* 30 min/day at 60–70% maximum workload on 3 days/week *Energy Restricted Diet* + *Control Exercise Group*: reduced energy intake by 1000–1500 kcal/day + 30 min/session at light intensity on 3 days/week *Energy Restricted Diet* + *Vigorous Intensity Group*: reduced energy intake by 1000–1500 kcal/day + 30 min/session at 60–70% maximum workload on 3 days/week	Oral glucose tolerance test	*Fasting Glucose* Vig. intensity groups had greater decrease compared to light-intensity groups
						*Fasting Insulin* Significant decrease in Energy Restricted Diet + Vig. Intensity group
						*Glucose AUC* Significant decrease in Energy Restricted Diet + Vig. Intensity group Vig. intensity groups had greater decrease compared to light-intensity group
						*Insulin AUC* Significant decrease in both Energy Restricted Diet groups Vig. intensity groups had greater decrease compared to light-intensity group Energy restriction groups had greater decrease compared to maintain diet groups
Larson-Meyer et al. [82]	48	25 ≤ BMI ≤ 30 kg/m^2^ Men (25–50 years) and women (25–45 years) Nonsmoking Exercise ≤ twice per week	6-month caloric restriction and aerobic exercise intervention	*Weight Maintenance Control Group* *Caloric Restriction Group:* 25% calorie deficit *Caloric Restriction* + *Exercise:* 12.5% calorie intake deficit plus 12.5% increase in exercise energy expenditure *Very low-calorie diet:* 890 kcal/day intake until 15% weight reduction followed by weight maintenance diet	Intravenous glucose tolerance test	*Fasting Glucose* No change
						*Fasting Insulin* Significant decrease in all calorie deficit groups
						*Insulin Sensitivity Index* Significant decrease in Calorie Restriction plus Exercise and Very Low-Calorie Diet groups
						*AIRg* Significant decrease in all calorie deficit groups
Bouchonville et al. [20]	107	BMI > 30 kg/m^2^ > 65 yrs Weight stable previous 12 months Physically inactive with mild-to-moderate physical frailty	12-month caloric restriction and aerobic exercise intervention	*No Exercise or Diet Control Group* *10% Diet-Induced Weight Loss Group*—prescribed calorie deficit of 500–750 kcal/day *Exercise Training without Weight Loss Group*—~ 90 min/session (30 min of balance and flexibility, 30 min of aerobic exercise at 70–85% peak HR, 30 min of 1 to 2 sets of 6 to 8 repetitions at 70–85% 1 RM of 9 resistance exercises *Diet* + *Exercise Group*—prescribed calorie deficit of 500–750 kcal/day + ~ 90 min/session (30 min of balance and flexibility, 30 min of aerobic exercise at 70–85% peak HR, 30 min of 1 to 2 sets of 6 to 8 repetitions at 70–85% 1 RM of 9 resistance exercises	Oral glucose tolerance test	*Insulin Sensitivity Index* Significant increase in Diet group and Diet + Exercise group Significant difference in change between Diet, Exercise, and Control groups vs. Diet + Exercise group
						*Insulin AUC* Significant decrease in Diet group and Diet + Exercise group Significant difference in change between Diet vs. Control and Exercise vs. Diet + Exercise
						*Glucose AUC* Significant decrease in Diet and Diet + Exercise groups Significant difference in change between Diet vs. Control and Exercise vs. Diet + Exercise
						*HOMA-IR* Significant decrease in Diet and Diet + Exercise groups Significant difference in change between Exercise vs. Diet + Exercise
Gilbertson et al. [19]	29	Average BMI: 35.9 kg/m^2^ 18 to 71 yrs Prediabetes Sedentary	16-week caloric restriction and aerobic exercise intervention	*Diabetes Prevention Program* + *Vigorous Intensity Interval Training*: energy restricted diet designed to induce 7% weight loss + 10, 30-s sprints at maximal self-selected intensity (within 10 bpm of maximum HR or RPE 19–20) followed by 4-min active rest at 2.0 mph and 0% grade on 3 days/week *Diabetes Prevention Program* + *Moderate Intensity Continuous Training*: energy restricted diet designed to induce 7% weight loss + 60 min/session at 45–55% HR reserve on 3 days/week	Blood draw	*Fasting Glucose* Significant decrease in both groups over time
						*Hemoglobin A*_*1c*_ Significant decrease in both groups over time
						*Fasting Insulin* No significant changes
						*HOMA-IR* No significant changes
Brennan et al. [84]	84	BMI ≥ 30 kg/m^2^ 60 to 80 yrs Weight stable previous 6 months Physically inactive Nonsmoking Systolic BP < 150 mmHg and Diastolic BP < 95 mmHg	6-month caloric restriction and exercise intervention	*Health Education Control Group*—biweekly general health education seminars on medication on type 2 diabetes management *10% diet-induced weight loss group*—prescribed calorie deficit of 500–1000 kcal/day and low-fat diet *Diet* + *Exercise Group*—prescribed calorie deficit of 500–1000 kcal/day and low-fat diet + 45 min/session of aerobic exercise at 50–80% HR_reserve_ on 4–5 days/week for 6 months; starting at week 8, added 30 min/session of resistance exercise on 2 days/week (9 exercises focused on major muscle groups; 2–3 sets of 10–12 repetitions each)	Hyperinsulinemic–euglycemic clamp	*Fasting Glucose* No between-group differences in change
						*Fasting Insulin* Diet + Exercise group had significantly greater improvement than control group
						*Hemoglobin A*_*1c*_ Diet + Exercise group had significantly greater improvement than control group
						*Rate of Glucose Disposal Corrected for Plasma Insulin* Diet + Exercise group improved significantly compared to control After controlling for % weight loss, no significant between-group differences
						*Endogenous Glucose Production Suppression* No between-group differences in change

AUC, area under the curve; BP, blood pressure; BMI, body mass index; HR, heart rate; HOMA-IR, homeostatic model assessment of insulin resistance; 1 RM, 1 repetition maximum

Cox and colleagues investigated the independent and additive effects of 16 weeks of caloric restriction alone, exercise alone, and caloric restriction combined with exercise on glucose and insulin metabolism [18]. Sixty non-diabetic (normal or impaired glucose tolerance) men with overweight or obesity were randomized to either maintain or restrict their energy intake (reduce by 1000–1500 kcal/day; 15% protein, 30% fat, and 55% carbohydrates). Within each caloric restriction arm, the participants were further randomized to 3 days per week for 30 min of either: (1) light-intensity exercise control group (1 session of flexibility exercise and 2 sessions/week of stationary cycling against zero resistance); or (2) vigorous-intensity exercise group (stationary cycling at 60 to 70% of their maximum workload). Fasting blood samples and a two-hour 75 g OGTT were performed following an overnight fast. Vigorous exercise training exhibited an independent effect on the glucose and insulin responses to an OGTT. Further, the combination of caloric restriction and vigorous intensity exercise provided an additive effect on reductions in insulin AUC. Thus, both caloric restriction and exercise provide a potent strategy to reduce the risk of impaired glucose tolerance, insulin resistance, and diabetes in sedentary men with overweight or obesity.

Larson-Meyer and colleagues evaluated the effects of 6 months of calorie restriction—with or without exercise—on insulin sensitivity, *β*-cell function, and body fat indices in the Comprehensive Assessment of the Long-Term Effects of Reducing Intake of Energy (CALERIE™; Phase 1 conducted at Pennington Biomedical Research Center) randomized controlled trial [82]. Forty-eight non-diabetic adults with normal glucose tolerance and overweight were randomly assigned to one of four groups: (1) control (weight maintenance diet); (2) calorie restriction (25% caloric restriction of baseline energy requirements); (3) calorie restriction plus exercise (12.5% caloric restriction and 12.5% increase in energy expenditure through structured exercise); or (4) very low-calorie diet (890 kcal/day caloric intake until 15% weight reduction followed by weight maintenance diet). The structured exercise program included 5 days per week of walking, running, cycling, or stairclimbing. Participants completed three sessions per week under supervision; the two unsupervised sessions were verified with portable heart rate monitors. The amount of time needed to expend the 12.5% calorie target was determined on an individual basis by calculating the oxygen cost of three self-selected exercise workloads on a treadmill, stationary cycle, or stairmaster [83]. To assess insulin sensitivity, three-hour IVGTTs were performed during inpatient stays at baseline and the end of the intervention following an overnight fast and at least 48 h after the final exercise session. Following the 6-month intervention, no group experienced a significant change in fasting glucose, whereas all three interventions significantly decreased fasting insulin and AIRg. Both the calorie restriction plus exercise and very low-calorie diet groups significantly improved insulin sensitivity index by 37 ± 18% and 70 ± 34%, respectively, while the calorie restriction alone group tended to increase insulin sensitivity index (40 ± 20%; *p* = 0.08); there were no significant differences among the three intervention groups. Improvements in insulin sensitivity index were correlated with reductions in weight, fat mass, and visceral adipose tissue, but not with adipocyte size or ectopic fat distribution in liver or muscle. Findings from this study suggest that when the energy deficit is held constant, calorie restriction alone or with aerobic exercise similarly improves skeletal muscle insulin sensitivity and early phase *β*-cell function for adults with overweight and normal glucose tolerance. Of note, participants in this study self-selected their exercise intensity, which may underestimate the role of exercise when combined with caloric restriction [83].

Gilbertson and colleagues examined the effect of a 16-week Diabetes Prevention Program combined with high-intensity interval training or moderate-intensity continuous training on glycemic control in sedentary adults with prediabetes [19]. Participants (*n* = 29) partook in the Diabetes Prevention Program and were randomized to three days per week of either: (1) high-intensity interval training (progressed to ten, 30-s sprints at maximal self-selected intensity within 10 bpm of HR_max_ or RPE of 19–20, followed by a 4-min active rest at 2.0 mph and 0% grade); or (2) moderate-intensity continuous training (progressed to 60 min of walking at 45–55% HR_reserve_). Fasting blood was drawn to assess glucose, insulin, HOMA-IR, and HbA_1c_. Both the high-intensity interval training and moderate-intensity continuous training groups significantly decreased fasting glucose (− 0.09 ± 0.01 and − 0.18 ± 0.02 mmol/L, respectively) and HbA_1c_ (− 0.21 ± 0.09 and − 0.12 ± 0.12%, respectively), with no difference between groups. Thus, when combined with the Diabetes Prevention Program, both exercise interventions effectively improved fasting glucose and HbA_1c_ among individuals with prediabetes.

Bouchonville and colleagues tested the independent and combined effects of weight loss and exercise on insulin sensitivity and other cardiometabolic risk factors in frail, older adults with obesity [20]. One hundred seven individuals were randomized to either (1) control group; (2) 10% caloric restriction-induced weight loss group (prescribed calorie deficit of 500–750 kcal/day and dietary intake of 1 g/kg of body weight of high-quality protein); (3) exercise training without weight loss (~ 90 min/session consisting of 30 min of balance and flexibility exercises, 30 min of aerobic exercise at 70–85% peak HR, and 30 min of resistance exercise of 1–2 sets, 6–8 repetitions at 70–85% 1 repetition maximum of 9 exercises); or (4) combined weight loss plus exercise group (full caloric deficit prescription plus a full exercise prescription) for 1 year. A standard 75 g OGTT with blood sampling was performed following an overnight fast. Insulin sensitivity increased in both weight loss only and combined weight loss plus exercise groups, with a greater improvement in the combined weight loss plus exercise group. However, no responses in insulin sensitivity were observed in either the exercise training without weight loss or control groups. The combined weight loss plus exercise and weight loss groups had similar improvements in insulin and glucose AUCs, with no changes observed in the exercise without weight loss or control groups. Thus, weight loss improves insulin sensitivity and other cardiometabolic risk factors, while an even greater improvement in insulin sensitivity can be achieved when exercise training is added to weight loss.

In a 6-month randomized controlled trial, Brennan and colleagues investigated the effects of caloric restriction-induced weight loss with and without exercise on insulin sensitivity in physically inactive older obese adults with or at high risk for type 2 diabetes [84]. Eighty-four participants were randomized to either (1) health education control group; (2) 10% caloric restriction-induced weight loss group (prescribed calorie deficit of 500–1000 kcal/day and a low-fat diet); or weight loss plus exercise group (full calorie deficit prescription plus a progressive exercise training program). The exercise program included 4–5 days/week (180 min/week total) of semi-supervised aerobic exercise performed at 50–80% HR_reserve_. Starting at week 8, participants were also prescribed 2 days/week (30 min/session) of resistance training with machines focused on major muscle groups. Markers of insulin sensitivity were assessed with a hyperinsulinemic–euglycemic clamp performed after an overnight fast. Compared to the health education control group, only the weight loss plus exercise group experienced a greater improvement in fasting insulin and HbA1c. After controlling for circulating insulin concentrations, only the weight loss plus exercise group improved peripheral insulin sensitivity—assessed as rate of glucose disposal accounting for plasma insulin during steady state—compared to the health education control group. However, after controlling for % weight loss, no significant between-group differences existed for peripheral insulin sensitivity. Further, no between-group differences were found in fasting glucose and endogenous glucose production. The authors noted that the caloric restriction-induced weight loss alone group experienced modest improvements in insulin sensitivity, while also experiencing reductions in lean mass and muscle strength. However, when exercise was added to caloric restriction-induced weight loss, more robust improvements occurred for skeletal muscle insulin sensitivity with concurrent maintenance of muscle mass and strength and reductions in ectopic fat deposition.

### Summary

As compared to caloric restriction or structured exercise alone, clinical lifestyle interventions clearly produce superior effects on markers of glycemic status derived from multiple techniques across the cardiometabolic disease spectrum. However, of the studies included in this narrative review, the extreme heterogeneity of study designs and populations preclude the ability to determine whether there is an optimal combination of different exercise and weight loss prescriptions. Although outside the scope of this review, disentangling the effects of various dietary strategies (e.g., nutrient timing and composition) further complicates the caloric restriction component [85].

Although little research exists investigating the underlying mechanisms of clinical lifestyle interventions, one possible explanation for superior effects of combined caloric restriction and exercise interventions on glycemic status relates to fatty acid oxidation and intramuscular lipid content [86]. Obesity is associated with a lower distribution of type I muscle fibers, impaired fatty acid oxidation, and increased lipid deposition. Weight loss effectively reduces skeletal muscle lipid content and increases skeletal muscle insulin sensitivity by increasing enzymes responsible for phosphorylation, storage, and oxidation of glucose, increasing GLUT4 expression, and increasing tyrosine kinase activity of skeletal muscle insulin receptors [86]. As mentioned above, aerobic exercise improves skeletal muscle insulin sensitivity and increases fatty acid oxidation, which may serve as a protective mechanism against the accumulation of intramuscular fat content.

Another benefit of clinical lifestyle interventions is that caloric restriction appears to independently improve free fatty acid-induced hepatic insulin resistance. As compared to 12 weeks of aerobic exercise training alone, combining aerobic exercise with 500 kcal/day of caloric restriction improved hepatic insulin sensitivity—measured during lipid-infused conditions of a two-stage euglycemic–hyperinsulinemic clamp—to a greater extent in adults with impaired glucose tolerance and obesity [87]. Thus, as lipid accumulation in the liver is a primary driver of obesity-related insulin resistance and type 2 diabetes [88], caloric restriction beneficially affects an additional target organ, creating a more potent benefit of clinical lifestyle intervention [85].

## Conclusion

As the disturbances in glucose homeostasis become more challenging to reverse along the progression to type 2 diabetes [5, 6], identifying the optimal preventive treatment early in the development of disease is an important public health matter. Overall, evaluation of results from the studies included in this narrative review highlights the following consistent findings: (1) randomized trials comparing combinations of aerobic and resistance exercise to either mode alone show combination training produces the greatest beneficial effects for insulin sensitivity and glucose homeostasis markers—in fact, the effects of the combination seem to be more than additive, implying that synergic mechanisms may be in play; and (2) lifestyle interventions incorporating weight loss via caloric restriction with aerobic exercise are superior to either lifestyle change alone, especially among individuals further along the progression to diabetes. Although existing evidence regarding amount and intensity of aerobic exercise is somewhat conflicting, moderate intensity—when matched for amount relative to body weight—appears to elicit the most beneficial improvements in IVGTT-derived skeletal muscle insulin sensitivity and early phase pancreatic *β*-cell function, as well as OGTT-derived glucose tolerance compared to vigorous intensity. On the other hand, for peripheral insulin sensitivity measures derived from hyperinsulinemic–euglycemic clamps, vigorous intensity exercise appears to elicit greater improvements. These concepts are also highlighted throughout the American College of Sports Medicine’s recently updated consensus statement regarding exercise and physical activity for individuals with type 2 diabetes [89].

The summation of evidence presented in this review also sheds light on key differences in study design further complicating the ability to derive a consensus for the optimal exercise exposure to improve glycemic status. As few true dose–response exercise trials exist to date, the heterogeneous study designs described herein reveal the difficult nature of discerning the optimal exercise prescription for prevention and treatment of type 2 diabetes. Although not completely understood, several mechanisms have been described for how aerobic exercise improves skeletal muscle, hepatic, pancreatic, adipose tissue, and whole-body insulin sensitivity. However, the physiologic pathways by which combining aerobic exercise with either resistance training or caloric restriction-induced weight loss produce their marked effects on insulin sensitivity remain elusive. As the public health burden of diabetes continues to grow, further investigation is critical to identify optimal exercise intervention characteristics focusing on combinations of mode, intensity, and amount for disease prevention and treatment.

## Data Availability

Data sharing is not applicable to this narrative review.

## Abbreviations

AMPK: Adenosine monophosphate-activated protein kinase
ATP: Adenosine triphosphate
AIRg: Acute insulin response to glucose
AUC: Area under the curve
DARE: Diabetes aerobic and resistance exercise trial
DI: Disposition index
GLUT4: Glucose transporter type 4
HART-D: Health benefits of aerobic and resistance training in individuals with type 2 diabetes trial
HbA_1c_: Hemoglobin *A*_1c_
HOMA-IR: Homeostatic model assessment of insulin resistance
HR: Heart rate
IVGTT: Intravenous glucose tolerance test
KKW: Kcal of exercise expenditure/kg of body weight/week
LP-IR: Lipoprotein Insulin Resistance Index
mTOR: Mechanistic target of rapamycin
OGTT: Oral glucose tolerance test
Si: Insulin sensitivity index
STRRIDE: Studies of a targeted risk reduction intervention through defined exercise trial
